# Clinical course and challenging management of early COVID-19 infection after heart transplantation: case report of two patients

**DOI:** 10.1186/s12879-021-05793-6

**Published:** 2021-01-20

**Authors:** Vincent Tchana-Sato, Arnaud Ancion, Julien Tridetti, Natzi Sakalihasan, Marie Pierre Hayette, Olivier Detry, Philippe Delvenne, Philippe Amabili, Marc Senard, Olivier Hougrand, Delphine Szecel, Jean-Paul Lavigne, Elie Minga Lowampa, Charlotte Ponte, Isabelle Maquoi, Philippe Morimont, Melissa Van Den Bulck, Marie Helene Delbouille, Jean Olivier Defraigne, Patrizio Lancellotti

**Affiliations:** 1 Department of Cardiovascular Surgery, CHU Liege, B35 SART TILMAN, 4000 Liege, Belgium; 2 Department of Cardiology, CHU Liege, Liege, Belgium; 3 Department of Clinical Microbiology, CHU Liege, Liege, Belgium; 4 Department of Abdominal Surgery and Transplantation, CHU Liege, Liege, Belgium; 5 Department of Pathology, CHU Liege, Liege, Belgium; 6 Department of Anesthesiology, CHU Liege, Liege, Belgium; 7 Department of Intensive Care, CHU Liege, Liege, Belgium

**Keywords:** Severe acute respiratory syndrome coronavirus 2 (SARS-Cov-2), Coronavirus disease 2019 (COVID-19), Heart transplantation, Asymptomatic carrier, Case report

## Abstract

**Background:**

There are limited data on Coronavirus disease 2019 (COVID-19) in solid organ transplant patients, especially in heart transplant recipients, with only a few case reports and case series described so far. Heart transplant recipients may be at particular high risk due to their comorbidities and immunosuppressed state.

**Case presentation:**

This report describes the clinical course and the challenging management of early COVID-19 infection in two heart transplant recipients who tested positive for the SARS-CoV-2 virus in the perioperative period of the transplant procedure. The two patients developed a severe form of the disease and ultimately died despite the initiation of an antiviral monotherapy with hydroxychloroquine coupled with the interruption of mycophenolate mofetil.

**Conclusions:**

These two cases illustrate the severity and poor prognosis of COVID-19 in the perioperative period of a heart transplant. Thorough screening of donors and recipients is mandatory, and the issue of asymptomatic carriers needs to be addressed.

**Supplementary Information:**

The online version contains supplementary material available at 10.1186/s12879-021-05793-6.

## Background

In the current context of coronavirus disease 2019 (COVID-19), the management of patients with end-stage heart failure on the transplant waiting list, and of heart transplant recipients (HTR) is challenging. To date, only a few case reports or small case series of COVID-19 in HTR have been described in China, Spain, Germany, and the United States, with mainly HTR who were remote from transplant and on chronic immunosuppressive drugs [1–8]. The limited data published so far have shown that the clinical presentation of COVID-19 in those patients did not differ from non-transplant patients or other solid organ transplant recipients [1, 2, 5, 6, 8]. However, the case fatality rate in some case series varied from 23% to up to 33% [6, 8, 9]. Newly HTR constitute a particularly vulnerable cohort for severe COVID-19 infection due to high levels of immunosuppression and frequent comorbidities. Unfortunately, the clinical characteristics and the management of COVID-19 in this subgroup of patients remain largely unknown. Herein, we report on two HTR who developed and ultimately died from severe COVID-19 a few days after their urgent transplant procedure (TP). Both were completely asymptomatic at their admission in our center with no respiratory complaints. These cases highlight the clinical course of these two patients and the difficulties encountered in their management.

## Cases presentation

### Recipient 1 (Fig. 1)

On March 17, 2020, a 59-year-old man with end-stage ischemic heart disease was admitted for heart transplant after being on the waiting list for approximately 12 months. He was obese and had a history of mitral annuloplasty surgery 13 years earlier. The last weeks before admission, his clinical condition had worsened with rhythmic instability due to recurrent tachycardia treated by internal electric shocks on several occasions. At home and upon admission, there were no obvious fever or respiratory symptoms. Laboratory findings did not reveal any particular anomaly with a normal hemogram, renal function and C-reactive protein (CRP) (4.4 mg/L, nl: 0–5). The cross match with the donor was negative. Given the COVID-19 pandemic, he was screened for severe acute respiratory syndrome coronavirus 2 (SARS-CoV-2) infection by reverse transcriptase polymerase chain reaction (RT-PCR) assay targeting E and RdRP genes on nasopharyngeal swab (NPS) [10]. The donor was also screened for SARS-Cov-2 infection and was negative.

**Fig. 1:**
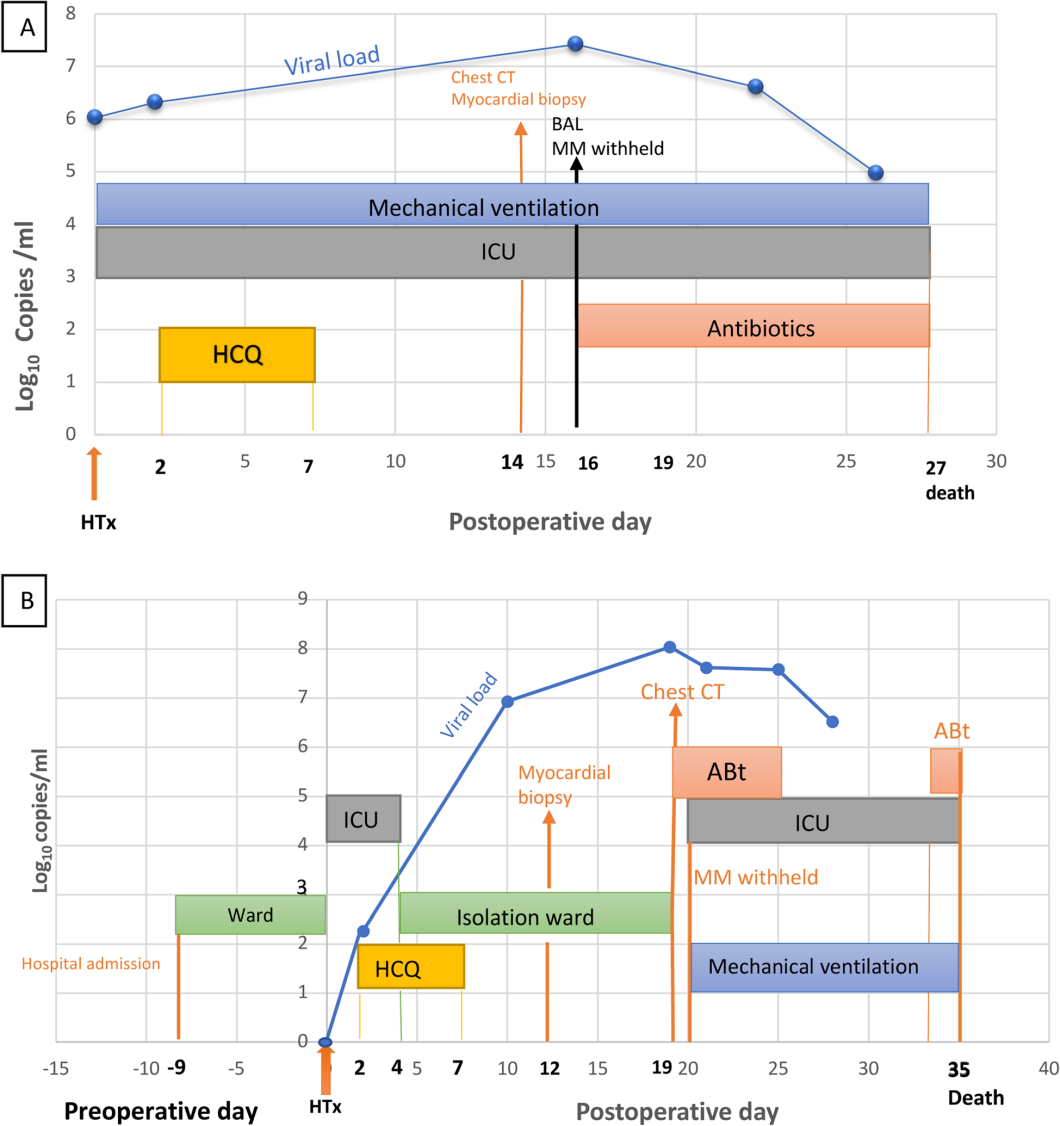
Clinical course of Recipient 1 (**a**) and Recipient 2 (**b**). From admission and day of surgery (HTx) to death highlighting the SARS-CoV-2 RNA load (Log_10_ copies/ml), the ward and ICU length of stay, diagnostic and therapeutic interventions such as chest CT, myocardial biopsy, BAL, initiation of antibiotics, and HCQ. **a** MM was withheld on POD 16. The clinical deterioration observed on POD 14 was close to the peak of the viral load observed on POD 16. **b** MM was withheld on POD 20. We can see that the deterioration of the patient’s clinical status on POD 19 coincides with a peak of the viral load. The SARS-CoV-2 RNA load is expressed by log_10_ copies per milliliter. Two RT-PCR assays were used due to a change in the testing method of the laboratory. The first target is the E and RdRP gene (Corman V.) The second test is the automated Cobas© SARS-CoV-2 molecular test (Cobas 6800 Roche) targeting the E and ORF1ab genes. Only the E gene is illustrated in the figure. ABt, antibiotics; BAL, bronchoalveolar lavage; Chest CT, chest computed tomography; HTx, heart transplantation; HCQ, hydroxychloroquine; ICU, intensive care unit; MM, mycophenolate mofetil; POD, postoperative day; RT-PCR, reverse transcriptase polymerase chain reaction

The graft was transplanted in the orthotopic position. He was easily weaned off cardiopulmonary bypass (CPB) and transferred to the intensive care unit (ICU) on mild inotropic support. Quickly after his arrival in the ICU, he became hemodynamically unstable despite adequate filling and required an escalation of inotropic and vasopressor support. Upon arrival in the OR and after performing a transesophageal echocardiography (TEE), a cardiac tamponade was excluded, and it was decided to go for a surgical revision due to stenosis of the superior vena cava anastomosis. At the end of the intervention, the patient who regained hemodynamic stability was transferred to the ICU.

The induction therapy included a standard triple regimen of decreasing doses of methylprednisolone, a calcineurin inhibitor (tacrolimus) to obtain trough levels between 8 and 10 μg/L, and an antimetabolite, mycophenolate mofetil (MM) 1000 mg twice daily. Anti-infectious prophylaxis included sulfamethoxazole 800 mg and trimethoprim 160 mg three times a week and valganciclovir hydrochloride 450 mg once a day.

The results of the preoperative SARS-CoV-2 RT-PCR analysis returned positive on POD 2. Therefore, according to the hospital protocol, the patient received 200 mg of hydroxychloroquine (HCQ) twice daily from POD 2 to POD 7 after a loading dose of 400 mg twice daily.

**Fig. 2:**
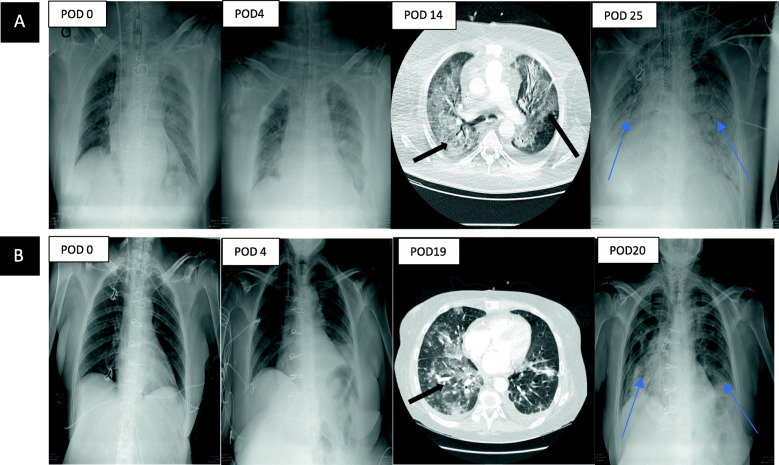
Chest X-ray and CT of recipient 1 (**a**) and recipient 2 (**b. a**) Progressive occurrence of pulmonary lesions during the postoperative course. The black arrows illustrate the ground-glass opacity lesions compatible with COVID-19 infection on POD 14. The blue arrows illustrate bilateral parenchymal patchy consolidations at POD 25. **b** Progressive occurrence of pulmonary lesions during the postoperative course until readmission in the intensive care unit on POD 20, where we can clearly see the ground-glass opacities lesions compatible with COVID-19 infection at POD 19 (black arrow), and bilateral patchy consolidations at POD 20 (blue arrow). POD, postoperative day

The immediate postoperative period was marked by the occurrence of right ventricular dysfunction and low cardiac output syndrome requiring a further increase in the doses of dobutamine and norepinephrine, as well as the administration of inhaled nitric oxide to overcome refractory hypoxia. He developed acute renal failure requiring renal replacement therapy. The situation gradually improved until POD14, when the respiratory exchanges started to deteriorate, and chest computed tomography (CT) revealed bilateral ground-glass opacities compatible with a COVID-19 infection (Fig. 2). On the same day, right cardiac catheterization demonstrated mild postcapillary pulmonary hypertension with a slightly reduced cardiac index. The myocardial biopsy did not demonstrate cellular or humoral rejection. However, analysis by electron microscopy revealed viral-like particles within endothelial cells but not in cardiomyocytes.

Bacterial infection was confirmed by the isolation of *Klebsiella oxytoca* in bronchoalveolar lavage (BAL) (> 10^6^ CFU/ml) and in blood cultures. The patient was treated with cefepime for 10 days. SARS-coV-2 RT-PCR performed on BAL fluid revealed a high viral load (7.4 log10 copies/mL). At that time, MM was withheld, and methylprednisolone was reduced to 16 mg daily. Unfortunately, the patient’s respiratory exchanges continued to deteriorate with ineffective prone positioning. Venovenous extracorporeal membrane oxygenation (ECMO) was not considered in this immunosuppressed patient with more than 14 days of mechanical ventilation. He died on POD 27 of refractory hypoxia and multiorgan failure. Additionally, the surgeon tested positive for SARS-CoV-2 on POD 5.

### Recipient 2 (Fig. 1)

The second patient was a 56-year-old woman with decompensated hypertrophic heart disease who had been on the waiting list for more than 12 months. Her past medical history included surgery for left pulmonary vein stenosis in 2015. She did not have anti-HLA antibodies. At the time of transplant, she was in the cardiology ward on inotropic support for a new episode of heart failure. Indeed, in the past 3 months, she had been admitted three times for recurrent episodes of heart failure treated with dobutamine. In the ward, she had no fever or symptoms suggestive of respiratory infections. The biology performed before the heart transplant demonstrated normal white blood cell count (WBC) (6.15 10^3^/mm3; nl: 4.6–10.10), slight anemia (Hg 10 g/dL, nl: 11.7–15), mild renal failure (creatinemia 1.11 mg/dL; nl 0.55–1.02), and inflammatory syndrome (CRP 15.6 mg/L; nl 0–5). The cross match with the donor was negative. As the first recipient, she benefitted from systematic SARS-CoV-2 screening by RT-PCR on NPS 2 days before the TP.

TP was performed with a donation after circulatory death (DCD) heart procurement procedure as described by our group [11]. The donor tested negative for SARS-CoV-2. After retrieval, the heart graft was transported to a contiguous OR where the aforementioned TP, as well as the re-exploration just a few hours earlier, were carried out. Usual OR cleaning and changing of the whole respiratory circuit as well as the CO_2_ sampling line were performed between the two procedures. The graft was transplanted in the orthotopic position. She was easily weaned off CPB and transferred to the ICU.

The induction therapy and anti-infectious prophylaxis were the same as for the first recipient. The preoperative SARS-CoV-2 RT-PCR performed on NPS was negative. However, given the patient’s indirect contact with the first recipient through OR, another NPS was performed on POD 2. The result was reported to be weakly positive the same day. Therefore, from POD 2 to POD 7, the patient received 200 mg of HCQ twice daily, after a loading dose of 400 mg twice daily.

**Fig. 3:**
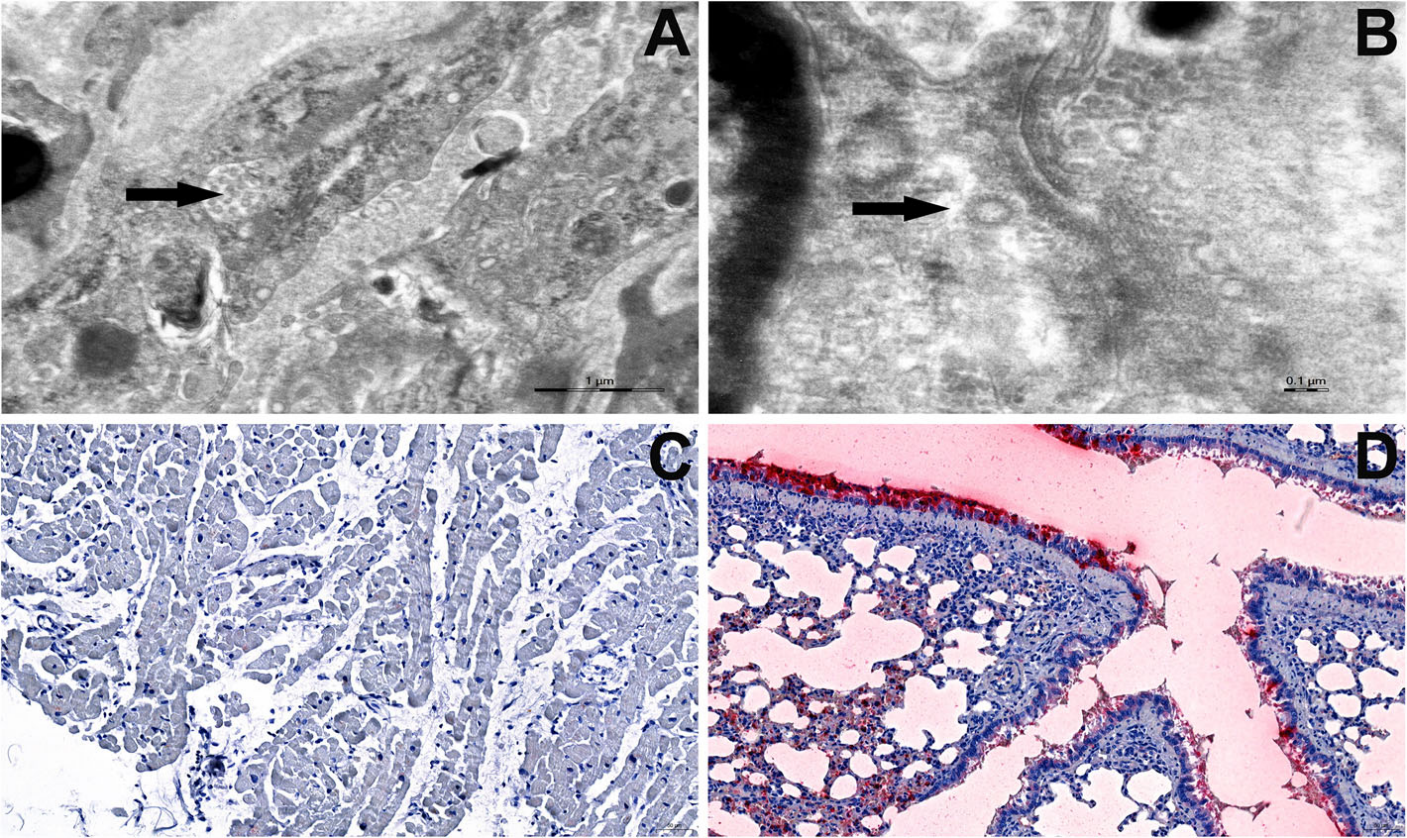
Electron microscopy (**a-b**) and immunohistology (**c-d**) of heart tissue from recipient 2. **a-b** Transmission electron microscopy representative examples demonstrating particles whose morphology is compatible with coronavirus particles (arrows). **c-d** Absence of immunoreactivity for the viral nucleocapsid protein (**c**) which contrasts with the intense staining in the positive control (lung tissue specimen of a SARS-CoV-2 infected hamster) (**d**). Transmission electron microscopy. Tissues were fixed at 4 °C in 4% glutaraldehyde (Laborimpex, Brussels, Belgium) in phosphate buffer at pH 7.4 and postfixed in 1% osmium tetroxide (Laborimpex, Brussels, Belgium) for 1 H at 4 °C. They were then dehydrated in graded (70, 90, 100%) ethanol solutions (VWR International, Leuven, Belgium) and propylene oxide (Laborimpex, Brussels, Belgium), embedded in epon (SERVA, Zandhoven, Belgium) and hardened at 60 °C. Semi-thin sections were stained with 0.5% toluidine blue and examined by light microscopy. Ultra-thin sections (80 nm) were stained with uranyl acetate (Fluka, Bornem, Belgium) and lead citrate (Leica, Aartselaar, Belgium). These sections were examined using an EM 910 transmission electron microscope (60 kV) (Zeiss, Belgium). Immunohistology Sections were deparaffinized and rehydrated in graded alcohols. After endogenous peroxydase inhibition with H2O2 (3%), nonspecific binding sites were blocked with the Protein Block Serum-Free solution (Dako). Slides were incubated for 1H at room temperature with a rabbit polyclonal antibody against the SARS-CoV-2 recombinant fusion nucleoprotein (ABclonal). Immunodetection was performed with the Polyview plus AP-Rabbit and alkaline phosphatase (Enzo Life Sciences)

There were no postoperative complications. Consequently, the patient was discharged from the ICU and transferred to an isolation ward on POD4. The right heart catheterization assessment was unremarkable, and the myocardial biopsy did not show rejection. As for the first patient, analysis by electron microscopy showed the presence of viral-like elements within endothelial cells but not in cardiomyocytes (Fig. 3). However, histological signs of endotheliitis or viral antigen immunoreactivity were not observed in the biopsy specimen (Fig. 3).

Her clinical course was uneventful until POD 19, when she became febrile, and described breathing difficulties with a gradual decrease in her oxygen saturation requiring nasal oxygen supplementation. The chest CT revealed some ground-glass opacities lesions predominant on the right lung (Fig. 2). SARS-CoV-2 RT-PCR performed on BAL fluid tested strongly positive (8 log10 copies/mL). Despite any evidence of infection, empirical antibiotic therapy based on azithromycin was initiated for 7 days following recommendations of the infectious disease team. After progressive worsening of her respiratory status, the patient was readmitted to the ICU on POD20, where prompt invasive ventilation coupled to prone positioning was required. At that time, MM was withheld. ECMO was not considered in the context of immunosuppression with more than 10 days of mechanical ventilation. Her clinical status continued to decline. *Klebsiella pneumoniae* was identified in few quantity (10^4^ copies/ml) by PCR (filmArray pneumonia panel) on bronchial aspirate on POD 34, but was not found on standard Gram staining and culture which only revealed contamination by oropharyngeal flora. However, given the clinical context (recent TP, rising CRP and WBC), the patient was treated with meronem following recommendations of the infectious disease team. She passed away on POD 35 from refractory hypoxemic respiratory failure.

## Discussion and conclusion

The global pandemic of COVID-19 has severely challenged the health care systems that face the battle to limit the spread of the SARS-CoV-2 while providing the treatment and care that will save lives. In most countries, cardiac surgery activity has been restricted to urgent or emergent cases, including heart transplantation. The safety of continuing this procedure during the current COVID-19 outbreak is, however, unknown. The literature on this topic is still scant. HTR appears to be at particular high risk for both acquisition of COVID-19 infection and progression to severe disease due to high rates of comorbidities, immunosuppression, and multiple healthcare contacts [12].

Li et al. were the first to report the clinical course of two HTR with COVID-19. However, TP was performed 32 and 194 months before the infection, respectively [1]. The two patients presented with variable levels of severity, one mild, the other more severe and requiring prolonged hospitalization. Both patients survived the infection. Fernando-Ruiz et al. reported the occurrence of COVID-19 among 4 HTR in a single center in Spain. The median interval from transplantation was 12.6 years (range, 8.7–17.9 years). One patient died 10 days after admission for respiratory failure, one patient was still in the ICU, and two patients survived and were discharged home [3]. Since these initial reports, other papers describing the clinical course of COVID-19 in HTR have been published from Germany and the United States. They also featured HTR who were remote from transplant and were mostly receiving relatively low-dose maintenance immunosuppression [2, 4, 5, 7–9]. Recently, Latif et al. described a single-center case series of 28 HTR with a confirmed diagnosis of COVID-19 in the United States. The median time from HT was 8.6 years, and the case fatality rate was 25% [6]. Singhvi et al. also described a single -center case series of 22 HTR in the disease epicenter in New York. The median time from HT was 4.6 years, and the overall mortality was 22.7%, reaching 26.3% in hospitalized patients [9]. Finally, Rivinius et al. reported 21 HTR with COVID-19 in a recent nation-wide survey of all HT centers in Germany during the first months of the pandemic. The overall mortality in their study was 33.3% [8].

**Fig. 4:**
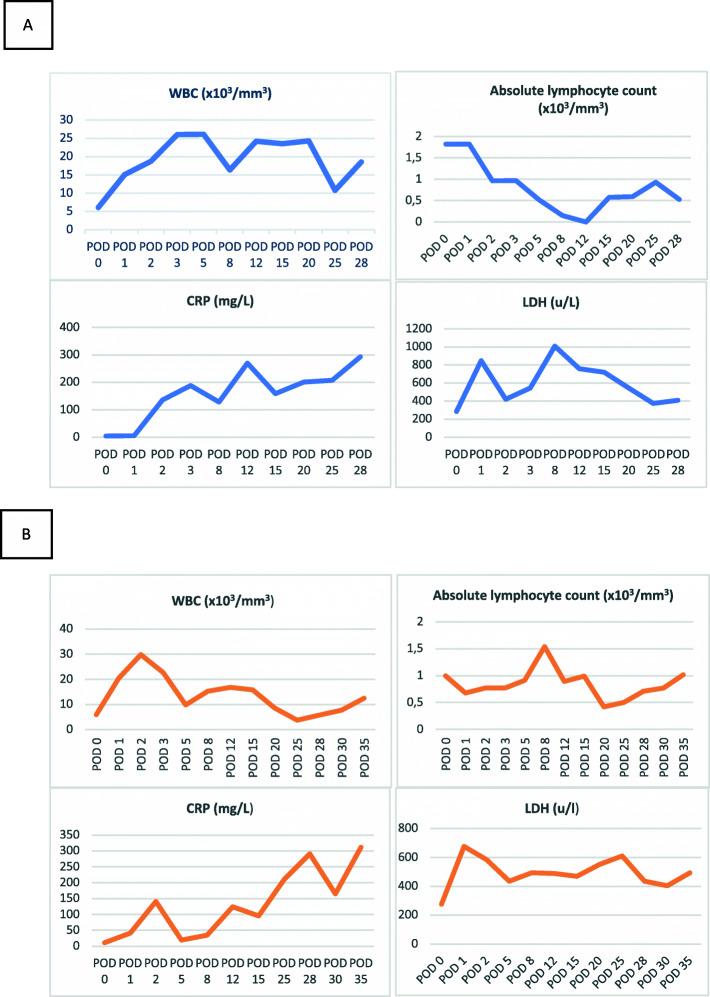
Laboratory findings (Blood count and inflammatory markers) of Recipients 1 (A) and 2 (B). CRP, C-reactive protein; LDH, Lactate dehydrogenase; WBC, White cell blood count

To our knowledge, our report is the first to highlight the clinical course of two HTR for whom viral carriage was confirmed by RT-PCR performed on NPS during the perioperative period. Both patients developed severe COVID-19 infection and ultimately died. The two patients had significant lymphopenia and elevation of inflammatory biomarkers such as CRP. However, the interpretation of the laboratory findings is complicated by confounding factors such as the recent transplant surgery, and the initiation of high doses of immunosuppressive drugs (MM). Laboratory findings are displayed in Fig. 4, Additional files 1 and 2, and Table 1. Histopathological analysis of the myocardial biopsy specimens of both patients did not demonstrate cellular or humoral rejection. However, analysis by electron microscopy revealed viral-like particles within myocardial endothelial cells but not in cardiomyocytes. SARS-CoV-2 gains entry to human cells by binding to the angiotensin-converting enzyme 2 receptor which is expressed in cardiac myocytes. In a case report of a 69-year-old patient with COVID-19 and cardiogenic shock, endomyocardial biopsy revealed viral like particles within interstitial cytopathic macrophages but not in cardiomyocytes or endothelial cells [13]. The coronary microvasculature and endothelium may be at risk for viral entry due to SARS-Cov-2 receptor expression on these cells. Indeed, Vargaz et al. found evidence of direct viral invasion of endothelial cells in several organs of patients with COVID-19 infection using electron microscopic images [14]. Both endotheliitis and cytokine release may play a major role in the pathophysiology of COVID-19 infection and may explain the association between cardiovascular disease and increased morbidity of this illness. In our patients, histological signs of endotheliitis or viral antigen immunoreactivity were not detected in the biopsy specimens suggesting a direct cytolytic effect of the virus unlikely but rather a pathophysiology mechanism based on cytokine release or immune response. This may result in systemic alteration of the microcirculatory function in different vascular beds, causing vasoconstriction, organ ischaemia, inflammation and a pro-coagulant state [14]. It is also likely that the underlying frailty of both patients, related to the heart failure condition, may have contributed to their unfavorable outcome.

**Table 1:**
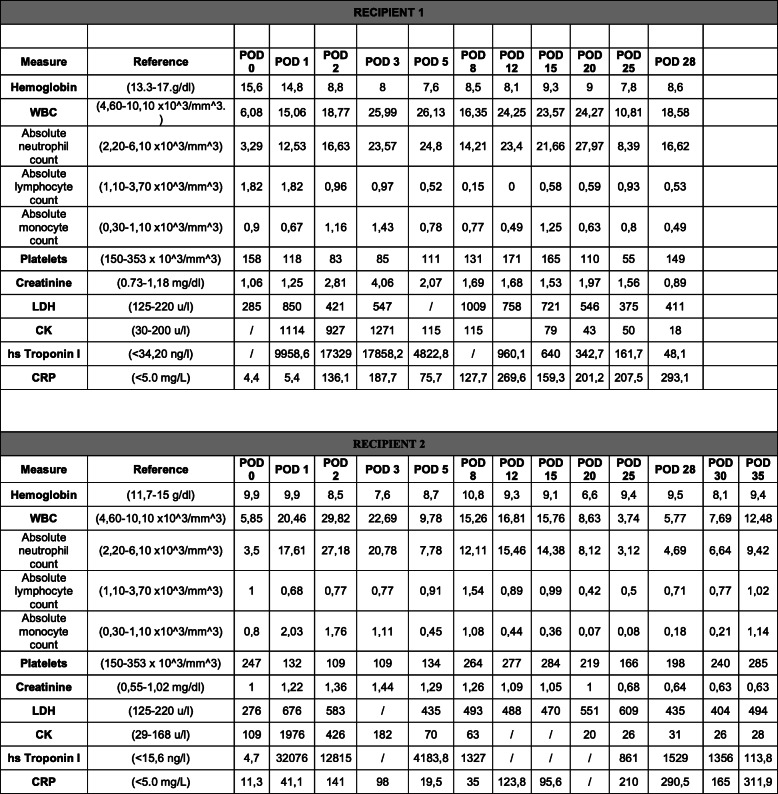
Laboratory studies of recipient 1 and recipient 2 throughout their clinical course

*CRP* C reactive protein, *CK* Creatinine kinase, *LDH* Lactate dehydrogenase, *hs* High sensitivity, *WBC* White cell blood count

Defining the precise mechanism of the viral transmission process in our report is challenging. At the time of both procedures, exposure investigations and contact tracing were not performed preoperatively. Recipient 1 was an unknown asymptomatic carrier before the surgery. It appeared later on that he lived in an area of high incidence for COVID-19 infection. Recipient 2 had been hospitalized for 9 days at the time of organ offer. Her preoperative SARS-CoV-2 screening test performed 2 days before the TP was negative, but she further tested positive on POD2. Transmission could theoretically have occurred through inhalation of the first recipient’s viral particles aerosolized in the OR. However, given the number of typical air changes per hour and the associated reduction in airborne contaminants, the possibility of OR transmission is relatively low [15]. Another possibility is contamination in the early preoperative period. The second recipient was never in contact with the first patient’s surgeon during her stay before transplantation. Among the health care workers (HCWs) with exposure to both patients from their hospital admission to their death, only the surgeon of the first procedure tested positive soon after the intervention. However, at that time, testing was only performed among HCWs who exhibited symptoms suggestive of SARS-Cov-2 infection; therefore, asymptomatic HCWs in close contact with both patients were not tested. After both HTR tested positive for SARS-CoV-2, inquiries were carried out among their close relatives and no cases of COVID-19 were reported. Consequently, the origin of viral transmission was never explained for recipient 2.

Practically, recently updated guidance documents from major transplant societies recommend not performing heart transplantation if the donor or the recipient is screened positive for SARS-CoV-2 [16, 17]. However, as illustrated in our cases, the lack of a rapid diagnostic test, the high proportion of asymptomatic carriers [18] capable of transmitting the infection among patients’ acquaintance, the high rate of false negative tests [19], the possibility of donor-recipient transmission, and the absence of massive COVID-19 testing all currently contribute to the impossibility of timely identification of patients who should not receive an emergency transplant.

The decision to perform two heart TP in the midst of a pandemic with no rapid preoperative testing available to rule out COVID-19 infection could be seriously questioned. However, at the time of both TP, all Belgian transplant centers were still pursuing their program. We were at the beginning of the pandemic in Belgium with 1486 COVID-19 confirmed cases and 14 deaths in the whole country (Additional file 3) [20]. There were 500 patients with COVID-19 hospitalized with 100 in the ICU in the whole country. In addition, our hospital’s prevalence of COVID-19 cases was low (28 patients). The two patients were transplanted just before the beginning of the nationwide lockdown (18th March 2020), and there was no indication that they were infected. At that period (17th March 2020), the ISHLT suggested proceeding with transplantation in waitlisted patients as long as there was no recent exposure or symptoms compatible with COVID-19 in the previous 2 weeks. Furthermore, RT-PCR for SARS-Cov-2 was also recommended depending on timing and testing availability [16]. Despite the fact that the first patient was called in from home, he had experienced recurrent episodes of life-threatening arrhythmia with a worsening of his clinical status the weeks before his transplant, and the second patient was in the cardiologic ward for a new episode of heart failure requiring inotropic support.

Questions remain on the optimal management of immunosuppression in transplant recipients with Covid-19. Current guidelines from expert associations recommend considering holding MM, mammalian target of rapamycin inhibitors or azathioprine in cases of moderate/severe COVID-19 infection, though specific data are still lacking at this time in the literature [16]. However, whether immunosuppression therapy can modify the course of the disease with either the benefit of a reduced immunological reaction or a greater risk of severe manifestation remains uncertain. In a literature review of published cases of COVID-19 in HTR performed at the beginning of the pandemic, Decker et al. found that immunosuppressive agents have been partially discontinued or reduced in dose in 71.8% of patients [21]. The elevated mortality rate in several case series of COVID-19 among HTR on chronic immunosuppressive therapy does not suggest a protective effect of the immunosuppressive drugs on the course of the infection [6, 8, 9]. However, further studies are needed to evaluate the impact of immunosuppressive therapy on the pathogenesis and outcomes of COVID-19 infection among HTR. In our two asymptomatic patients, we initially continued immunosuppressive drugs without any particular adjustment of the dose while prescribing HCQ known to improve viral clearance. Despite this, the evolution was unfavorable with the appearance of symptoms within 15 days, the standard incubation period for the virus. Even worse, the viral load remained high in both patients despite the interruption of MM in both cases. As confirmed by several recent clinical trials [22, 23], antiviral monotherapy with HCQ was ineffective in both cases. We did not observe any toxicity of that drug in either patient. The potential benefit of other antiviral and immunomodulator drugs has not been tested. The two patients eventually died. Mortality rates associated with COVID-19 increase sharply with age and in patients with underlying cardiovascular diseases such as heart failure [24–26]. Therapy for an overt disease is seriously lacking at the moment, although several promising molecules are still under active clinical investigations. Nonetheless, potential interactions between these medications and immunosuppressive drugs and the choice of the appropriate molecules according to clinical phenotype could add other challenges.

These two cases illustrate very well the severity and poor prognosis of COVID-19 infection in the immediate aftermath of a heart transplant. Moreover, our report highlights the problematic of asymptomatic carriers and of the delay in turnover time for COVID-19 testing results in HT. As transplant clinicians, we must be very careful about continuing the transplant program in this COVID-19 period. After these two cases, our heart transplant program was temporarily suspended. Our center has now increased its testing capacity as well as the use of serological testing. Our transplant policy is now in accordance with updated recommendations of major transplant societies. Donors and recipients are thoroughly screened to exclude infection and rule out any close contact with a person at risk or diagnosed with COVID-19 in the days preceding the transplant. The TP is not performed before obtaining the RT-PCR test results. In addition, after the procedure, patients should be regularly tested for COVID-19.

## Supplementary Information

**Additional file 1: Fig. 5-suppinfo** Evolution of the viral load and the absolute lymphocyte count in recipient 1

**Additional file 2: Fig. 6-suppinfo** Evolution of the viral load and the absolute lymphocyte count in recipient 2.

**Additional file 3: Fig. 7-suppinfo** Total confirmed COVID-19 deaths and cases in Belgium. (https://www.ecdc.europa.eu/en/covid-19-pandemic). The blue arrow represents the date of the two transplant procedures. The confirmed counts shown here are lower than the total counts. The main reason for this is limited testing and challenges in the attribution of the cause of death (Source: European CDC-Situation update Worldwide-Last updated 29th June, 11).

## Data Availability

The data that support the findings of this report are available from the corresponding author upon reasonable request.

## Abbreviations

COVID-19: Coronavirus disease 2019
CPB: Cardiopulmonary bypass
CRP: C reactive protein
CT: Computed tomography
DCD: Donation after circulatory death
ECMO: Extracorporeal membrane oxygenation
HCQ: Hydroxychloroquine
HTR: Heart transplant recipients
ICU: Intensive care unit
ISHLT: The international society for heart and lung transplantation
MM: Mycophenolate mofetil
NPS: Nasopharyngeal swab
OR: Operating room
POD: Postoperative day
RT-PCR: Reverse transcriptase polymerase chain reaction
SARS-coV-2: Severe acute respiratory syndrome coronavirus 2
TEE: Transesophageal echocardiography
TP: Transplant procedure
WBC: White blood cell count
