# Supplementary material for: Clinical course and challenging management of early COVID-19 infection after heart transplantation: case report of two patients
Source: BMC Infect Dis. 2021 Jan 20;21:89. doi: 10.1186/s12879-021-05793-6 (PMC7816134; doi:10.1186/s12879-021-05793-6)
Supplement: Supplementary file 1 — Additional file 1: Fig. 5-suppinfo Evolution of the viral load and the absolute lymphocyte count in recipient 1 [file 12879_2021_5793_MOESM1_ESM.pdf]

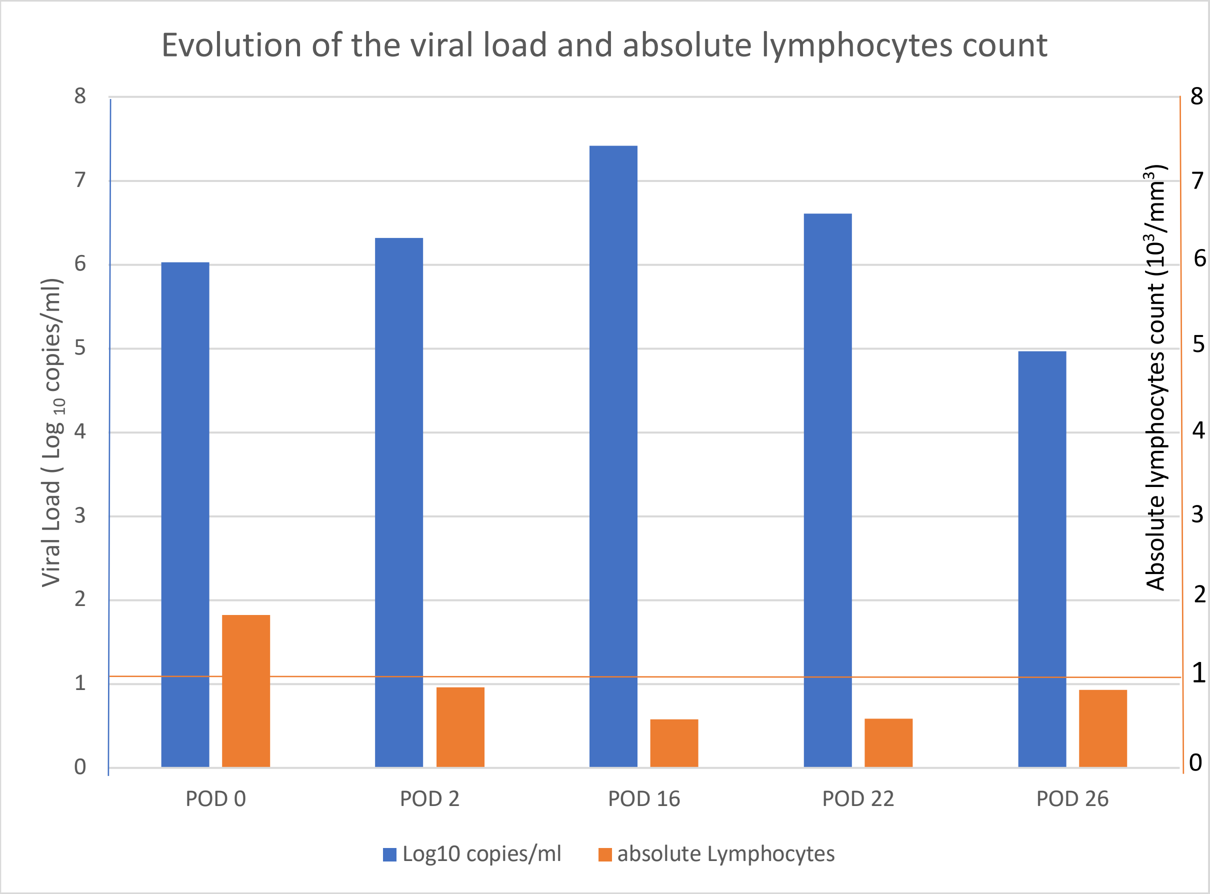

Fig.5 Evolution of the viral load (expressed by Log_{10} copies per milliliters) and the absolute lymphocytes count (expressed by $10^3/mm^3$) in **Recipient 1**

The orange horizontal line represents the inferior limit of the normal range of the absolute lymphocyte count. We can see a progressive lymphopenia while the viral load increases gradually until its peak on POD16.

POD,postoperative day

*The absolute lymphocyte count normal range (1,10-3,7x$10^3/mm^3$)
