# Supplementary material for: Clinical course and challenging management of early COVID-19 infection after heart transplantation: case report of two patients
Source: BMC Infect Dis. 2021 Jan 20;21:89. doi: 10.1186/s12879-021-05793-6 (PMC7816134; doi:10.1186/s12879-021-05793-6)
Supplement: Supplementary file 2 — Additional file 2: Fig. 6-suppinfo Evolution of the viral load and the absolute lymphocyte count in recipient 2. [file 12879_2021_5793_MOESM2_ESM.pdf]

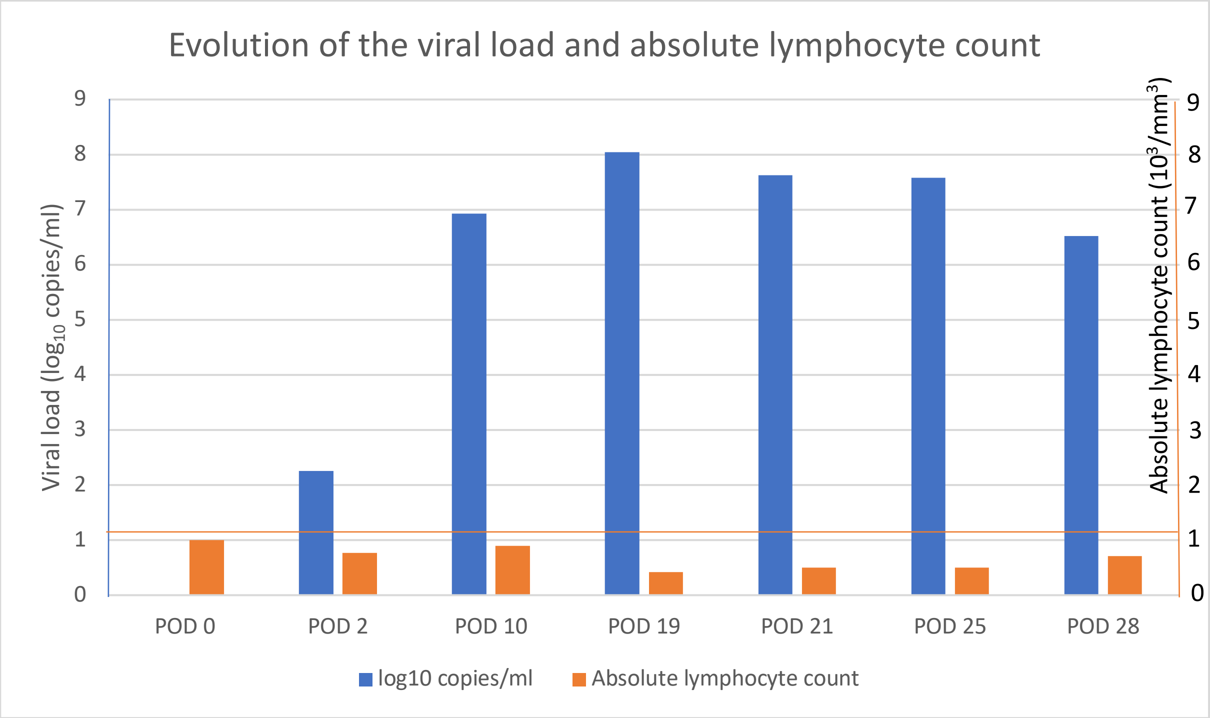

Fig.6 Evolution of the viral load (expressed by log_{10} copies per milliliters) and absolute lymphocytes count ($10^3/mm^3$) in **Recipient 2**

The Preoperative RT-PCR test was negative on POD 0. The horizontal orange line represents the inferior limit of the absolute lymphocyte count. We can observe a progressive lymphopenia while the viral load increases to reach a peak on POD 19.

POD, postoperative day; RT-PCR, reverse transcriptase polymerase chain reaction.

*Absolute lymphocyte count normal range (1,10-3,7.$10^3/mm^3$)
