# Supplementary figures and images for: Clinical course and challenging management of early COVID-19 infection after heart transplantation: case report of two patients
Source: BMC Infect Dis. 2021 Jan 20;21:89. doi: 10.1186/s12879-021-05793-6 (PMC7816134; doi:10.1186/s12879-021-05793-6)

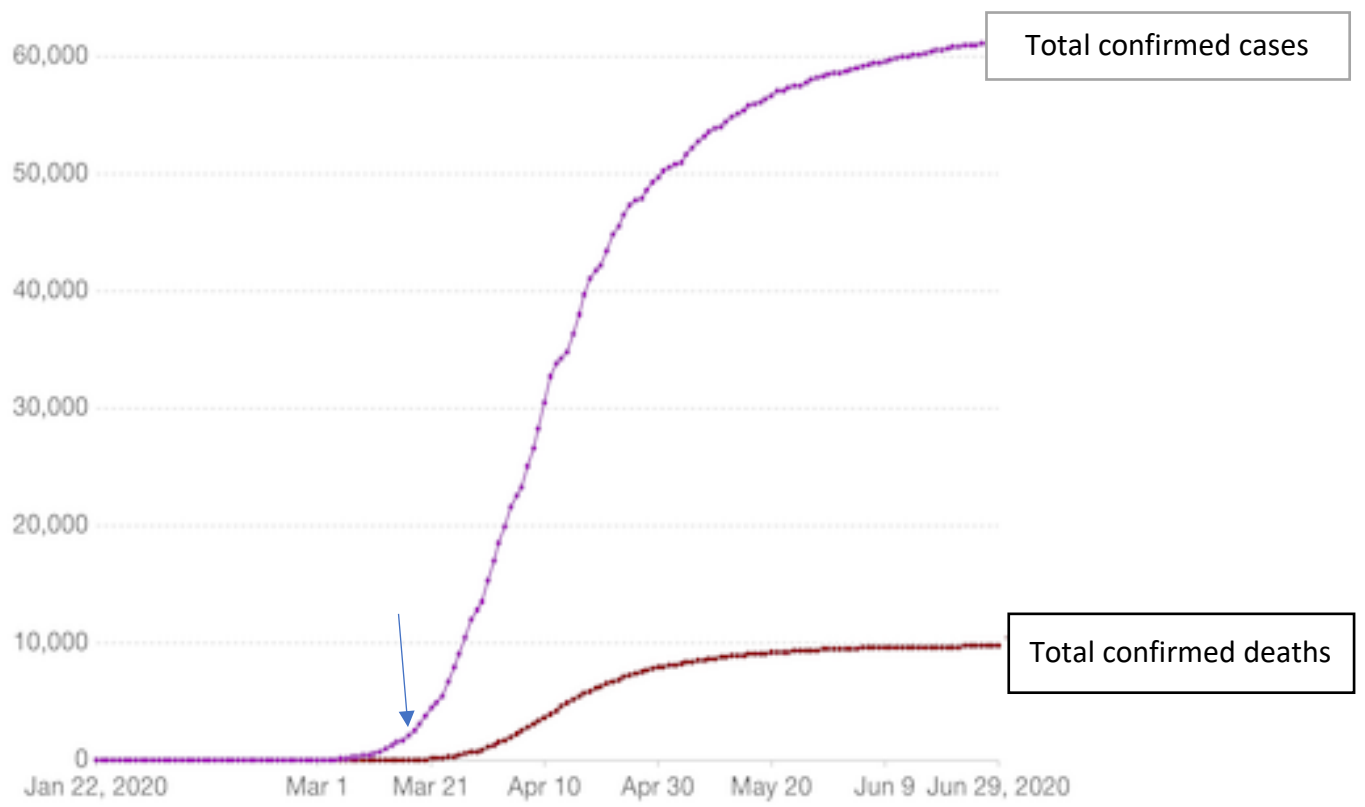

Fig.7 suppinfo

Supplement: Supplementary file 3 — Additional file 3: Fig. 7-suppinfo Total confirmed COVID-19 deaths and cases in Belgium. (https://www.ecdc.europa.eu/en/covid-19-pandemic). The blue arrow represents the date of the two transplant procedures. The confirmed counts shown here are lower than the total counts. The main reason for this is limited testing and challenges in the attribution of the cause of death (Source: European CDC-Situation update Worldwide-Last updated 29th June, 11). [file 12879_2021_5793_MOESM3_ESM.pdf]
